# Laughter as medicine: A systematic review and meta-analysis of interventional studies evaluating the impact of spontaneous laughter on cortisol levels

**DOI:** 10.1371/journal.pone.0286260

**Published:** 2023-05-23

**Authors:** Caroline Kaercher Kramer, Cristiane Bauermann Leitao

**Affiliations:** 1 Division of Endocrinology, University of Toronto, Toronto, Canada; 2 Leadership Sinai Centre for Diabetes, Mount Sinai Hospital, Toronto, Canada; 3 Lunenfeld-Tanenbaum Research Institute, Mount Sinai Hospital, Toronto, Canada; 4 Division of Endocrinology, Hospital de Clínicas de Porto Alegre, Porto Alegre, Brazil; 5 Post-graduate Program in Medical Sciences: Endocrinology, Universidade Federal do Rio Grande do Sul, Porto Alegre, Brazil; Houston Methodist Academic Institute, UNITED STATES

## Abstract

**Objectives:**

Laughter as an expression of humor has been recognized as *good medicine* for centuries. The health benefits of humor-induced well-being remain unclear and thus we conducted a systematic review and meta-analysis of interventional studies to evaluate the impact of spontaneous laughter on stress response as measured by cortisol levels.

**Design:**

Systematic review and meta-analysis.

**Data sources:**

MEDLINE/PubMed, EMBASE, PsycINFO, Scopus, and Clinicaltrials.gov.

**Eligibility criteria:**

Interventional studies, which could be either randomized placebo-controlled trials (RCTs) or quasi-experimental studies, conducted in adults that compared any spontaneous laughter intervention to a controlled setting and reported changes in cortisol levels were selected.

**Data extraction and synthesis:**

We examined the impact of laughter on percentage change in cortisol levels by calculating pooled estimates of the absolute differences between arithmetic means before and after interventions as compared to control using random-effects model.

**Results:**

Eight studies (315 participants; mean age 38.6) met our inclusion criteria; four were RCTs and four were quasi-experiment studies. Five studies evaluated the impact of watching a humor/comedy video, two studies evaluating laughter sessions administered by a trained laughter therapist, and one study evaluating a self-administered laughter program. Pooling these data showed a significant reduction in cortisol levels by 31.9% (95%CI -47.7% to -16.3%) induced by laughter intervention compared to control group with no evidence of publication bias (P = 0.66). Sensitivity analyses demonstrated that even a single laughter session induced a significant reduction of 36.7% in cortisol (95%CI -52.5% to -20.8%). In addition, analyses including the four RCTs reinforced these results by demonstrating a significant reduction in cortisol levels promoted by laughter as compared to the placebo arm [-37.2% (95%CI -56.3% to -18.1%)].

**Conclusions:**

Current evidence demonstrates that spontaneous laughter is associated with greater reduction in cortisol levels as compared with usual activities, suggesting laughter as a potential adjunctive medical therapy to improve well-being.

**Trial registration:**

**Registration number:** CRD42021267972.

## Introduction

Laughter as an expression of humor has been recognized as a *good medicine* for centuries [1], a concept consistent with human neurodevelopment. Specifically, the capacity for laughter in humans precedes the neural development of speech [2] with neuroimaging studies suggesting a unique neural pathway for spontaneous laughter (i.e. genuine laughter) [3] that is intuitive and subcortical. Documented in Charles Darwin’s classic work [4], ancestral forms of play homologous to human laughter have been reported in other animals (dogs, chimps, and even rats) [2, 5, 6] being perceived by Darwin and other authors as a natural response that restores homeostasis across diverse species including *homo sapiens*.

In 1976, a remarkable publication in *The New England Journal of Medicine* highlighted a potential therapeutic role for spontaneous laughter [7]. In that report, the author/patient described his regimen of self-prescribed laughter as an adjunctive therapy that provided him with potent anesthetic effect attenuating symptoms caused by ankylosing spondylitis: *“I made the joyous discovery that 10 minutes of genuine belly laughter had an anesthetic effect and would give me at least two hours of pain-free sleep”* [7]. Subsequently, recent research has linked laughter and humor with increased levels of pain tolerance [8], and positive impact in overall well-being in diverse medical settings such as oncology [9], psychiatry [10], and rehabilitation [11]. Data from a recent randomized controlled trial (RCT) demonstrated a positive and robust effect of having a humor journal on well-being [12], suggesting that humorous interventions are simple underutilized strategies that can help with coping in an adverse situation such as the coronavirus pandemic [13].

The activation of the hypothalamic-pituitary-adrenocortical (HPA) axis and consequent elevation in glucocorticoids is a key physiological response to either physical (i.e. acute and chronic diseases) or psychological stressors (i.e. anticipated recognition of a threat). Coupled with the autonomic nervous system, the HPA axis represents a vital neuroendocrine system that is dynamically regulated in a feedback-loop manner in order to maintain homeostasis of virtually the entire human body [14]. Despite the long-dated acknowledgment of humor as medicine, the impact of spontaneous laughter on neuroendocrine stress response remain unclear. Previous reports in adults have suggested that spontaneous laughter can impact the HPA axis by reducing cortisol levels [15–22]. However, the small sample sizes preclude definitive conclusions based on these individual studies, suggesting the need for a robust and systematic evaluation of the mechanistic effect of laughter by meta-analysis. Thus, we conducted a systematic review and meta-analysis of interventional studies to evaluate the impact of spontaneous laughter on cortisol levels.

## Methods

This systematic review and meta-analysis is reported in accordance with the Preferred Reporting Items for Systematic Reviews and Meta-Analyses (PRISMA) Statement and was registered with the International Prospective Register of Systematic Reviews (http://www.crd.york.ac.uk/prospero/; CRD42021267972) [23].

### Research ethics approval

Not applicable.

### Data sources and searches

We selected relevant studies published between 1950 and April 20, 2022. We searched MEDLINE/PubMed, EMBASE, PsycINFO, Scopus, and Clinicaltrials.gov using the following combined text and Medical Subject Heading (MeSH) terms: “laughter”, “laughter therapy”, “humor therapy”, “cortisol”. The complete PubMed search was as follows: ("Laughter"[Mesh] OR "Laughter therapy"[Mesh]) OR ("Laughter"[Text word] OR "Laughter therapy"[Text word]) OR (Humor therapy [Text Word]). The online Table 1 shows the complete search strategies used to search each database or registry. All potentially eligible studies were considered for review, regardless of the primary outcome or language. We also conducted a manual search using references of the included articles published in English.

**Table 1:** Characteristics of included studies.

Author (Year)	Study design	Population studied	Mean age (years)	% of men	Laughter intervention (setting)	Control group	Cortisol assay (no samples/time of collection)	% Cortisol reduction (95%CI) [absolute Δ change]	Conclusion
Berk et al (1989)	Quasi-experimental	10 healthy men (5 intervention and 5 control)	30.9	100	60 min humor video followed by 30 min recovery (group setting)	No stimuli	Serum cortisol (3–6 per time point/late afternoon)	-31.7% (95%CI -62.9 to -0.6) [Δ -30 nmol/l]	Mirthful laughter experience reduced cortisol levels
Vlachopoulos et al (2009)	Randomized controlled trial	18 healthy individuals (cross-over:18 intervention and 18 control)	26.9	50	30 min humor movie (not available)	Sitting quietly	Serum cortisol (not available)	-37.2 (95% CI -44.4 to -29.9) [Δ -1.67 μg/dl]	Laughter decreases cortisol levels
Bains et al (2015)	Randomized controlled trial	30 (20 healthy older adults and 10 individuals with diabetes) (20 intervention and 10 control)	68.5	56.6	20 min humor video (individual setting)	Sitting quietly	Salivary cortisol (2 per time point/10:30 am to 4 pm)	-15.0% (95%CI -37.5 to 7.5) [Δ -0.02 μg/dl]	Watching humor video reduced cortisol levels with borderline P value
Heo et al (2016)	Quasi-experimental	40 hemodialysis patients (20 intervention and 20 control)	53.4	42.5	4 sessions of 60 min laughter sessions by a trained laughter therapist + daily 15s laughter phone calls (4 weeks in total) (combined group and individual settings)	No stimuli	Serum cortisol (1 per time point/8 am)	-13.8% (95%CI -44.0 to 16.4) [Δ -0.54 μg/dl]	No significant differences were observed in serum cortisol levels
Fujisawa et al (2018)	Randomized controlled trial	80 healthy university students (40 intervention and 40 control)	24.0	62.5	30 min humor video followed by 30 min recovery (group setting)	Reading a book	Salivary cortisol (1 per time point/not available)	-63.0% (95%CI -82.3 to -43.8) [Δ -28.3 nmol/l]	Comedy group exhibited a significant decrease in cortisol levels by time
Lee et al (2018)	Quasi-experimental	50 pre-menopausal women living with obesity (24 intervention and 26 control)	42.3	0	12 sessions of laughter therapy program over 6 weeks (group setting)	No stimuli	Serum cortisol (1 per time point/am)	-0.4% (95%CI -22.4 to 21.6) [Δ 0.03 μg/dl]	There were no significant differences in cortisol levels between the groups
Lee et al (2020)	Quasi-experimental	48 nursing students (24 intervention and 24 control)	Not available	12.5	6 laughter sessions by a laughter instructor of 60 min intervention (not available)	No stimuli	Salivary cortisol (1 per time point/am)	-70.4% (95%CI -104.6 to -36.3) [Δ-0.63 ng/mL]	A statistically significant improvement in salivary cortisol levels was observed after laughter therapy.
Froehlich et al (2021)	Randomized controlled trial	39 adults (20 intervention and 19 control)	24.1	30.3	9 min humor video followed by a stress test (not available)	Neutral video	Salivary cortisol (not available)	-21.0% (95%CI -74.4 to 32.4) [Δ -1 nmol/l]	Humor did not significantly affect salivary cortisol

### Study selection

**Fig 1:**
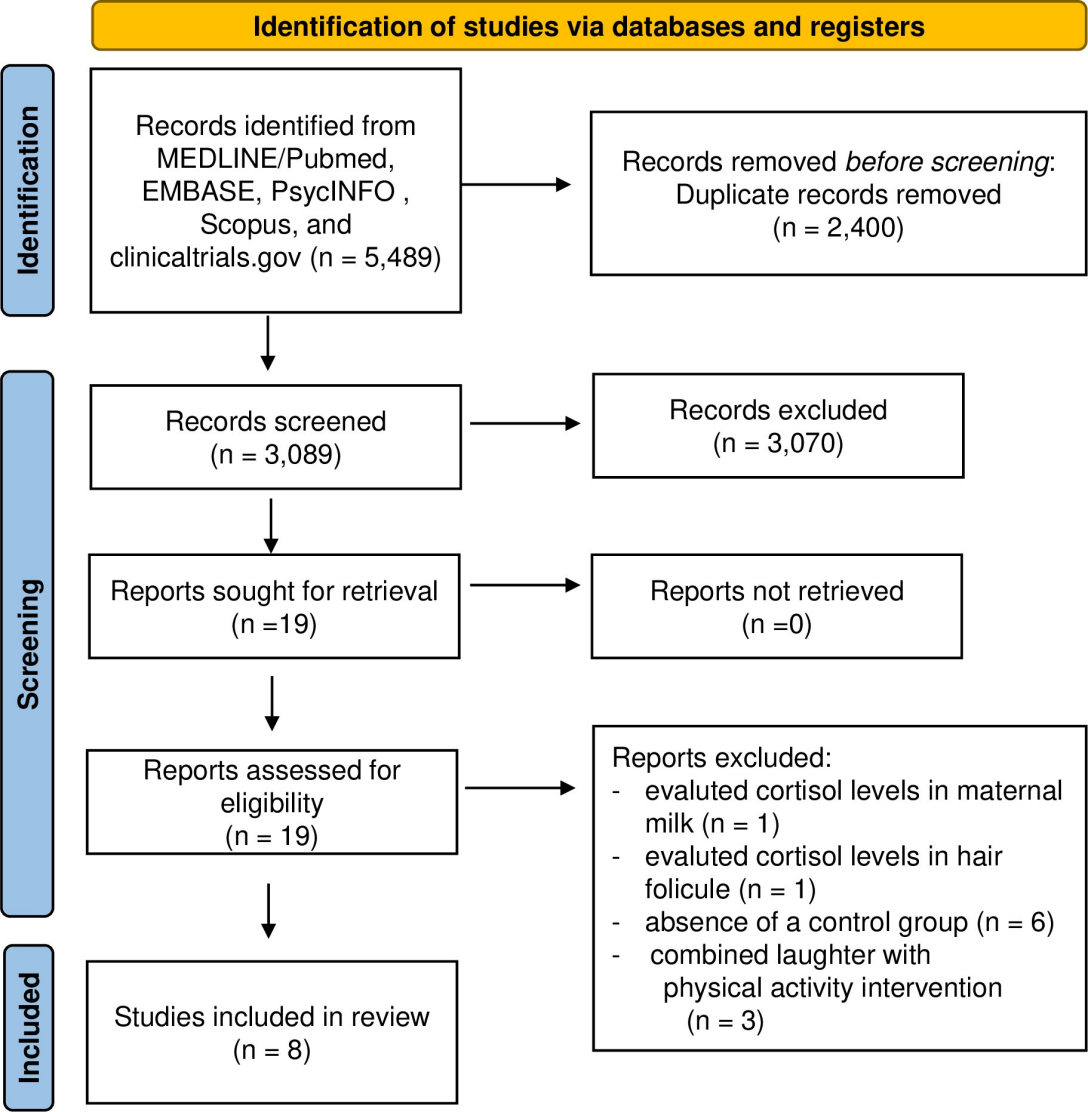
Flow diagram of the literature search to identify interventional studies evaluating laughter interventions on cortisol levels.

Studies were eligible for inclusion if they: (1) were interventional studies, which could be either RCT or quasi-experimental studies conducted in adults, (2) compared any spontaneous laughter intervention to a controlled setting consisting of either an intervention not associated with humor (i.e. such as reading a book) or usual activities (control group), and (3) reported changes in cortisol levels (serum/plasma or salivary cortisol). Exclusion criteria were as follows: studies that evaluated laughter-inducing therapy as part of physical activity intervention given the impact of exercise itself on stress hormones [24], studies that did not report a control group, retrospective studies or observational studies, and studies that measured cortisol in unstandardized samples (i.e. breast milk and hair follicle) (Fig 1). If a study reported data on more than one humorous intervention arm, we included the data that fulfilled our inclusion criteria.

### Intervention investigated and outcome measurements

We evaluated any spontaneous laughter intervention, which included (i) watching a comedy movie, (ii) laughter therapy conducted by a trained laughter therapist consisting of activities that promoted laughter, and (iii) self-administered laughter therapy. The laughter intervention could be applied on only one experimental day or as intermittent therapy sessions over a continuous period of time. The primary outcome was the percentage mean difference in the change in cortisol (serum/plasma or salivary) between baseline and end of intervention between intervention and control groups. These data was calculated based on changes in cortisol in relation to baseline values. The measure of variance was extracted by either absolute values, graphical displays, or estimated according to the Cochrane handbook of systematic reviews or meta-analysis.

### Data extraction and quality assessment

Two independent investigators (CKK, CBL) reviewed study titles and abstracts. Studies that satisfied the inclusion criteria were retrieved for full-text assessment. Studies selected for review by both investigators had an agreement value (k) of 97%; disagreements were resolved by further discussion between the investigators. The following data was extracted from each study: study design, total number of participants, duration of intervention, percentage mean changes in cortisol levels. The risk of bias was evaluated according to the revised Cochrane tool for assessing risk of bias in randomized trials (RoB 2) [25] and the risk of bias in non-randomized studies of interventions (ROBINS-I) tool [26]. In addition, we evaluated the quality of evidence using the Grading of Recommendations Assessment, Development and Evaluation (GRADE) for meta-analyses [27].

### Data synthesis and analysis

We examined the impact of spontaneous laughter on cortisol levels as assessed by percentage mean change in cortisol. We calculated pooled estimates of the absolute differences between arithmetic means before and after interventions as compared to control group using a random-effects model (inverse-variance DerSimonian-Laird method). The *I*^*2*^ value was used to evaluate the magnitude of heterogeneity between studies, with values greater than 50% indicating moderate-to-high heterogeneity [28]. We performed meta-regression analyses to assess whether the duration of laughter-inducing intervention impacted the stress response measured by changes in cortisol levels. In addition, we performed the following sensitivity analyses: (i) including studies that evaluated the impact of a single session of humorous intervention on cortisol levels, (ii) stratifying the studies by laughter intervention (watching comedy movie and laughter therapy), (iii) stratifying the studies by cortisol assay (salivary and serum cortisol), and (iv) including only RCTs. The possibility of publication bias was evaluated using a funnel plot of effect size against the standard error for each trial. Funnel plot asymmetry was evaluated by Begg’s and Egger’s tests, with significant publication bias defined as a *P* value <0.1 [29]. All analyses were performed using Stata 14.0 (Stata Corp, College Station, Texas).

### Patient and public involvement

There was no patient involved in this study design and results. However, we hope that with the publication of this report, further studies and implementation of interventions to improve well-being through laughter could benefit patients.

## Results

### Study characteristics

We identified 3,089 studies, of which 3,070 were excluded on the basis of title and abstract. Nineteen studies were retrieved for detailed assessment, eleven of which excluded (Fig 1). Eight studies (with data from 315 participants) met our inclusion criteria; four were RCTs [16, 17, 19, 22] and four were quasi-experiment studies [15, 18, 20, 21] (Table 1).

Included studies were published between 1989 and 2021, five studies evaluated laughter intervention as a single experiment [15–17, 19, 22] while three studies evaluated more than one laughter session over 4–6 weeks of time [18, 20, 21]. Patients had a mean age of 38.6 years old (ranging from 24.0 to 68.5 years), and 12.5–62.5% were male with one study conducted only in men [15] and one only in women [20]. The population evaluated in these studies included healthy individuals [15, 16, 19, 21, 22], participants living with diabetes and obesity [17, 20], and patients on hemodialysis [18]. The humorous intervention adopted in these studies varied, with five studies evaluating the impact of watching a humor/comedy video [15, 16, 17, 19, 22], two studies evaluating laughter sessions administered by a trained laughter therapist [18, 20], and one study evaluating a self-administered laughter program [21]. Salivary [17, 19, 21, 22] and serum [15, 16, 18, 20] cortisol were assessed at the same time of the day between the study arms, although the time of cortisol collection varied between the studies (Table 1).

**Fig 2:**
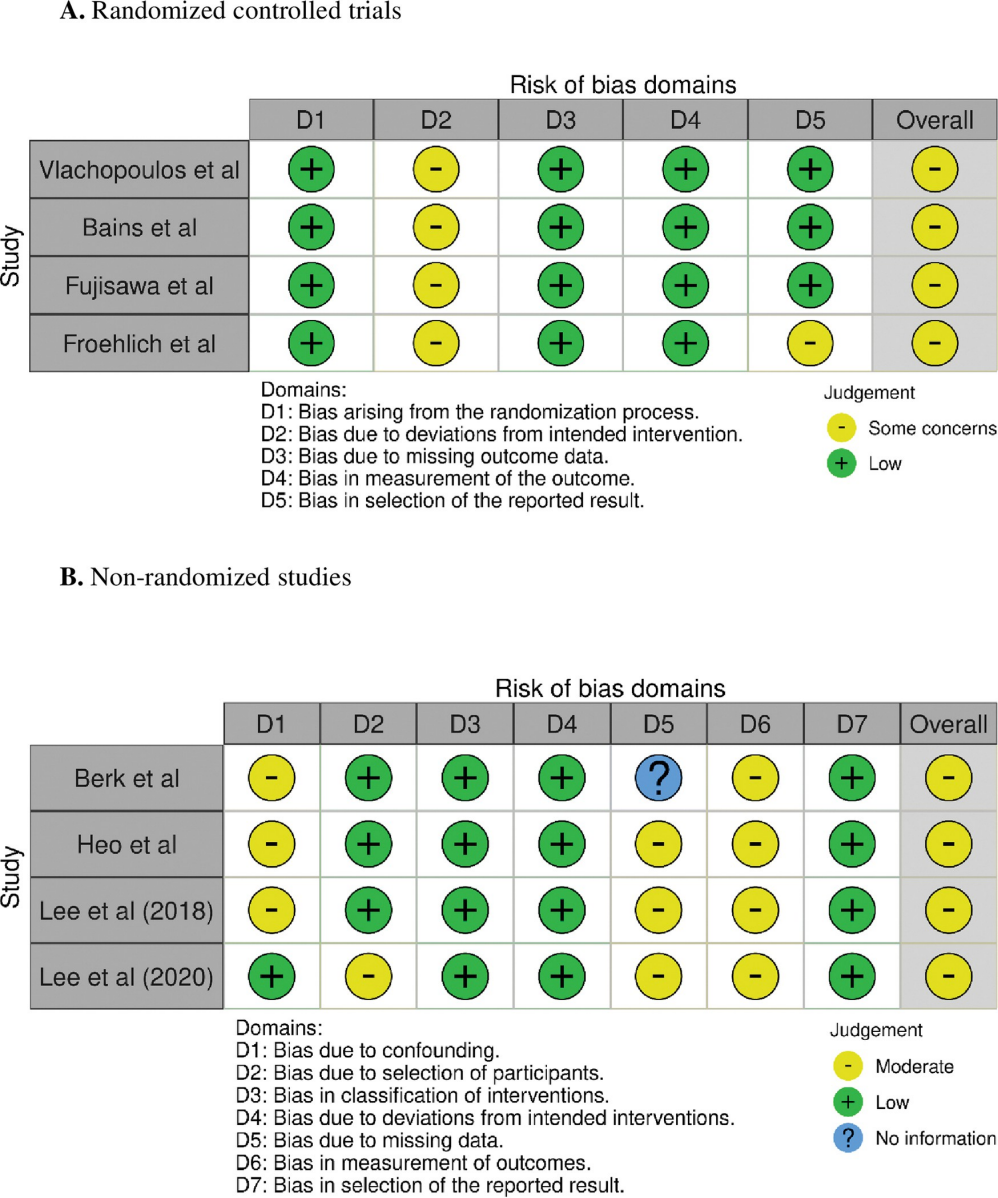
Assessment of risk of bias for each included study. (Panel A) Risk of bias in randomized trials (RoB 2). (Panel B) Risk of bias in non-randomized studies of interventions (ROBINS-I).

The assessment of risk of bias is shown in Fig 2. The RCTs presented concerns in the overall assessment of bias, mainly due to deviations on the intended intervention as the laughter intervention cannot remain blinded to the participants (Fig 2A). In the same way, the quasi-experimental studies had moderate possibility of bias related to possible confounding effect and impossibility of blinded intervention (Fig 2B).The dropout rates varied from 0% to 27.5%. Although some studies informed the participants about the possibility of adverse events with the humor intervention, there were no adverse events reported.

### Impact of laughter on cortisol levels

**Fig 3:**
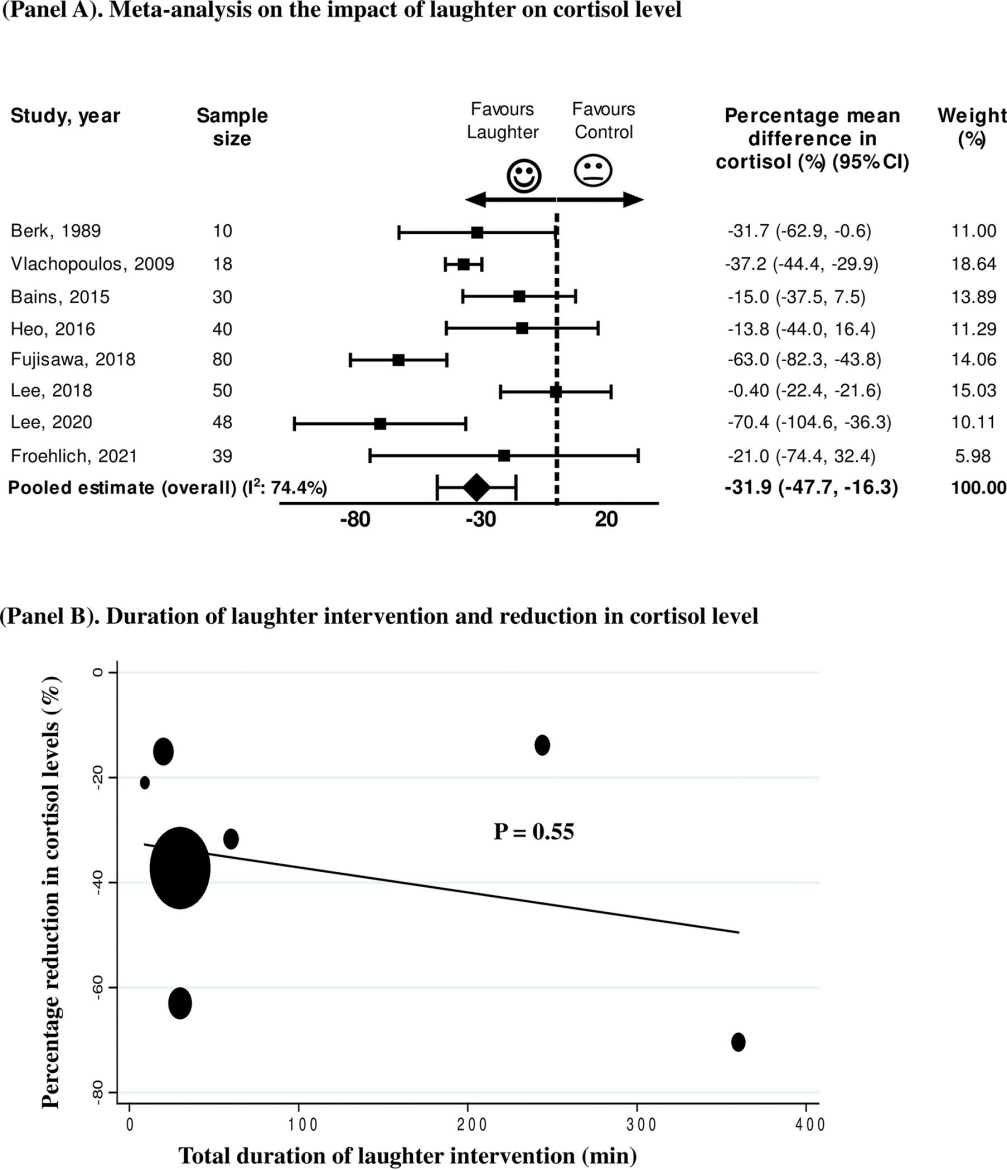
(Panel A) Meta-analysis of the percentage mean difference in cortisol (%) between laughter interventions and control. CI = confidence interval. (Panel B) Bubble plot with fitted meta-regression line showing relationship between duration of laughter intervention and reduction in cortisol.

Eight studies assessed the change in cortisol levels between laughter-inducing intervention and control group [15–22]. Pooling the data from these studies (n = 315 participants) showed a significant reduction in cortisol levels by 31.9% (95%CI -47.7% to -16.3%) induced by humorous intervention compared to control, with between-study heterogeneity (I^2^ 74.4%, P < 0.01) (Fig 3A). In this analysis, there was no evidence of publication bias on the Egger test (P = 0.66). In order to evaluate whether the duration of laughter-inducing activity (minutes) impacted the attenuation of stress response measured by reduction in cortisol levels, we performed meta-regression analyses which demonstrated no impact of the duration of spontaneous laughter on the observed reduction in cortisol (Fig 3B). To further assess the impact of laughter intervention on cortisol levels, we performed additional analysis including the studies that assessed a single laughter session (ranging from 9 to 60 minutes) [15–17, 19, 22]. This analysis demonstrated that even a single laughter session induced a reduction of 36.7% in cortisol (95%CI -52.5% to -20.8%) as compared to control group. In addition, analyses stratified by the type of laughter intervention (watching comedy movie and laughter therapy) demonstrated a reduction in cortisol level regardless of the strategy to induce spontaneous laughter [comedy movie: -36.7% (95%CI -52.5% to -20.8%); laughter therapy: -18.9% (95%CI -34% to -3.2%)].

**Fig 4:**
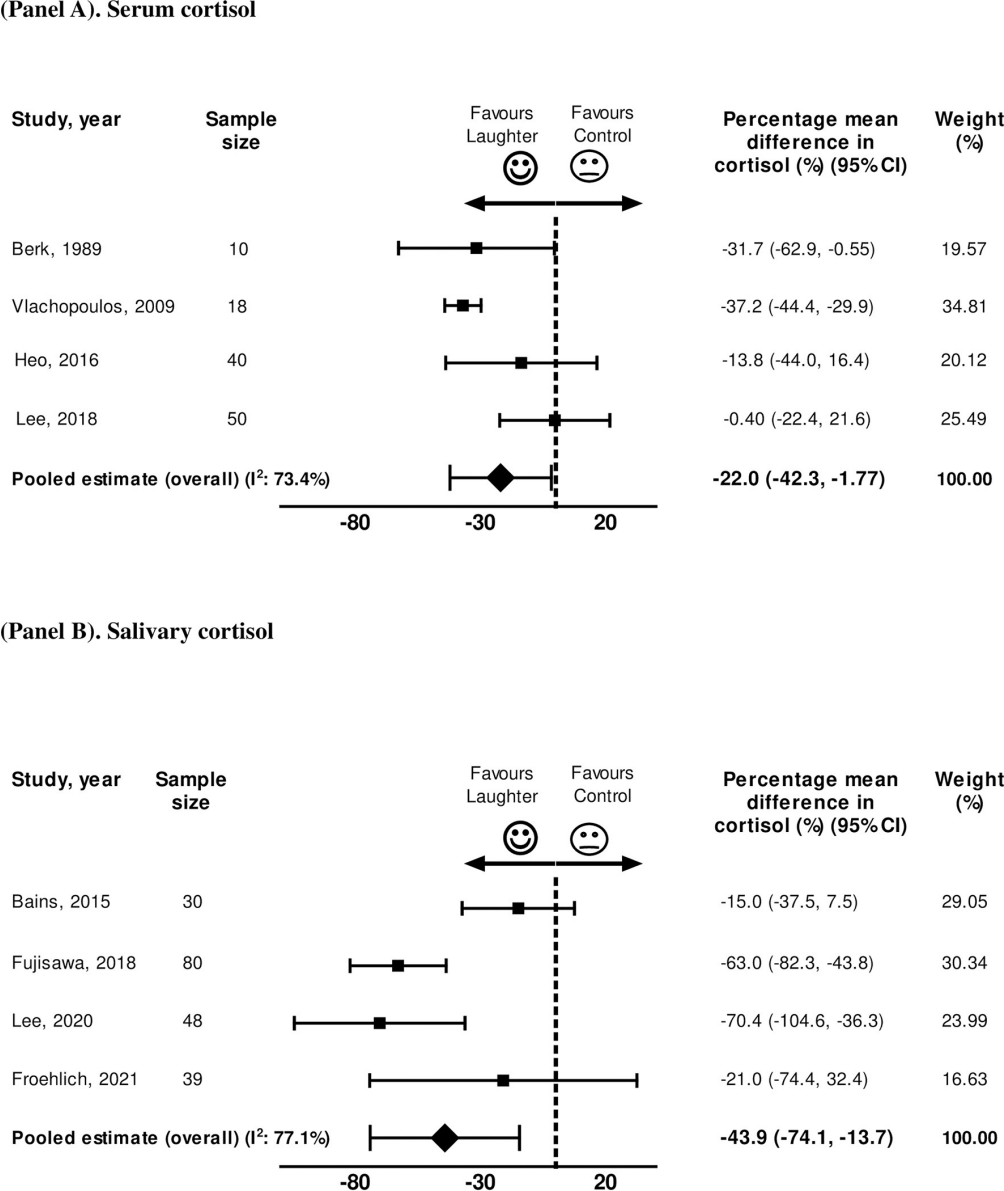
Meta-analysis of the percentage mean difference in cortisol (%) between laughter interventions and control. (Panel A) Serum cortisol, and (Panel B) Salivary cortisol. CI = confidence interval.

Next, sensitivity analyses were performed stratifying by the cortisol assay: (i) serum/plasma cortisol, and (ii) salivary cortisol measurements (Fig 4). As compared to control group, the laughter intervention induced significant decrease in cortisol in studies evaluating serum/plasma cortisol [-22.0% (95%CI -42.3% to -1.77%)] (Fig 4A). This effect was even more pronounced when the stress response was assessed by changes in salivary cortisol with a 43.9% reduction in cortisol induced by laughter as compared to control (95%CI -74.1% to -13.7%) (Fig 4B). Finally, we repeated the analyses restricting the analysis to the four RCTs [16, 17, 19, 22] which also demonstrated a significant reduction in cortisol levels promoted by laughter as compared to the placebo arm [-37.2% (95%CI -56.3% to -18.1%)]. The overall GRADE profile from these analyses is moderate/low (not free of biases) for establishing clinical recommendations based on the quality of the data available (Table 2).

**Table 2:** Grading of Recommendations Assessment, Development and Evaluation (GRADE) evidence profile from systematic review and meta-analysis of interventional studies evaluating impact of spontaneous laughter on cortisol levels.

No of studies (No of participants)	Study limitations	Consistency	Directness	Precision (cortisol measurement)	Publication bias	Relative effect (95%CI)	Overall Quality
8 (n = 315)	Moderate limitations	Unexplained heterogeneity	Direct	No important imprecision	Unlikely	-31.9 (95%CI -47.7 to -16.3%)	+++, moderate to low

## Discussion

Our results showed that, compared to non-humorous usual activities, interventions that trigger spontaneous laughter induced a significant reduction of ~32% in cortisol levels, suggesting an impact on HPA-axis as a metabolic pathway associated with the stress-relief effect of humor. The positive impact of laughter on buffering cortisol response was already evident after one single laughter session (~37% reduction), being observed regardless of the laughter-inducing strategy (watching comedy movie and laughter therapy) or cortisol assay (salivary and serum cortisol).

### Findings in the context of existing studies

Previous studies have demonstrated the potential impact of sense of humor and laughter on cardiovascular health [30–32]. Laughter-inducing activities such as watching a comedy movie has been associated with improvement in endothelial function assessed by brachial artery flow-mediated vasodilation and carotid arterial compliance, with effects that lasted up to 24-hours [30, 31]. The cardioprotective effect of positive emotions was also evident in clinical studies. Specifically, in a study of 300 individuals, Clark et al showed that the propensity to laugh under a variety of situations encountered in everyday life had an inverse association with incident coronary heart disease [32]. This concept is supported by epidemiological data from Women’s Health Initiative (n = 97,253 women) [33], Nurses’ Health Study (n = 69,744) [34], and the Veterans Affairs Normative Aging Study (n = 1,429 men) [34]. These studies demonstrated that optimism, which is closely related to spontaneous laughter (i.e. frequency of laughter is associated with optimism) [1–4], is inversely and independently associated with both cardiovascular and total mortality, being associated with exceptional longevity [33, 34]. Our results expand and reinforce the importance of spontaneous laughter in promoting health by demonstrating a significant and objectively-measured reduction on the hallmark adrenal stress-hormone, cortisol, induced by genuine laughter.

A reduction in cortisol levels has also been reported in response to laughter-inducing interventions in children and adolescents [35–39]. A previous meta-analysis of 24 studies (n = 1,612 kids) demonstrated that hospital clowns might contribute to improved psychological well-being and emotional responses in children and adolescents in hospital setting, with four studies reporting reduced levels of salivary cortisol after seeing hospital clowns visits compared with the pre-intervention measurement [35]. The implementation of humor in clinical care has been more widely established in the pediatric population possibly because the psychological motivations behind genuine laughter in adults is more complex, being influenced by the individual’s cultural background, education, beliefs, and psychological traits [1–4, 40]. Once developed, however, the interpretation and perception of laughter among adults overcomes cultural and language barriers. In a study evaluating 966 participants from 24 societies, individuals reliably distinguished friends from strangers based on acoustic features of laughter with an accuracy of 53–67% [41]. These results suggest that, despite the complexity of modern social interactions and diverse cultural backgrounds, laughter is still a nonverbal vocal expression that communicates positive affect and cooperative intent in humans. In parallel to that, laughter can be socially contagious as experiencing other people’s laughter is a powerful stimulus for further laughter [42] even if the joke lacks comedic component (i.e. a bad joke) [43].

The blunted response in cortisol induced by spontaneous laughter may have other positive metabolic effects beyond the most evident relaxation and well-being. Hayashi et al, evaluating the impact of laughter induced by a comedy show in individuals with type 2 diabetes not on insulin (16 men and 3 women, age 63.4 years, body mass index 23.5 kg/m^2^, glycated hemoglobin 7.2%) as compared to a usual lecture, observed that the humorous show reduced the postprandial glucose excursion by 2.5 mmol/l [44]. Another potentially desirable metabolic effect of laughter includes the energy consumption that results from the contraction of facial and abdominal skeletal muscles (diaphragm). In a study evaluating 55 healthy individuals (age 18–34 years; mean body mass index 24.7 kg/m^2^) using whole-room indirect calorimeter equipped with audio recording system, it was estimated that 15 minutes of genuine laughter can increase energy expenditure by approximately 40 kcal [45]. Interestingly and relevant to our results, reduced cortisol levels may help hair growth as evidenced by a recent study evaluating the underlying mechanism that modulates hair growth in response to stress. In a study published in *Nature*, Choi et al have uncovered a cellular and molecular mechanism that links stress hormones produced by adrenal glands to the activation of hair-follicle stem cells through the control of growth arrest-specific 6 (GAS6) expression in dermal papillae [46].

### Implications

The health benefits of self-induced or simulated laughter have also been reported previously [47] with the most common modality consisting of a combination of yoga breathing techniques with induced laughter exercises, the *laughter yoga*. We focused this meta-analyses on genuine laughter as (i) the majority of the human humor occurs spontaneously in everyday life [13], (ii) the combination of laughter with physical activity could impact the interpretation of the cortisol response as physical activity itself can impact the secretion of adrenal hormones [22], and (iii) previous imaging studies suggest the involvement of different neural pathways in stimulated as compared to spontaneous laughter [3]. The impact on HPA axis found in our analyses suggests that genuine laughter holds positive effects for overall health as the excessive/prolonged cortisol secretion associated with chronic HPA-axis stimulation has negative implications for both physical and psychological diseases including obesity, depression, and chronic pain [48, 49]. RCTs evaluating the long-term impact of genuine laughter are needed to establish whether activities that induce spontaneous laughter could be applied in specific clinical scenarios such as to improve metabolic health, symptoms of anxiety, and coping with chronic stressful situations.

### Potential limitations of this study

A possible limitation of our meta-analyses is the heterogeneity amongst the laughter-inducing protocols as the laughter interventions included watching movies and talking to a laughter therapist for different durations of time. In addition, the studies had small samples sizes. However, this is not expected to impact our conclusions as our results were consistent across the diverse protocols and even a single session of laughter was significantly associated with a decreased cortisol response. Another limitation pertains to the inherent bias of quasi-experiment studies design that cannot be ruled out. Nonetheless, the sensitivity analyses including only RCT’s reinforce the main results. In addition, despite the concerns observed in the assessment of risk of bias (Fig 2), the main aspect of concern related to the fact that laughter interventions cannot be applied in a blinded fashion. In this context, the potential impact of social interactions on HPA-axis response to laughter is another relevant aspect that could have impacted the results. Our analyses included studies with laughter intervention applied in both group and individual settings, but the degree of social interaction prior to or combined with the laughter intervention was not described. Another weakness is that the timing of cortisol sampling was inconsistent between studies and could have implications for the degree of change in cortisol induced by laughter. However, the fact that the timing of collection was not different between the study arms and that we assessed percent changes in cortisol may have helped to limit the impact of this possible confounder. Finally, pooling the data on the impact of laughter on cortisol levels in absolute units was not suitable given the diverse biochemistry measurements performed by each study.

## Conclusion

In conclusion, our results support the ancient knowledge that spontaneous laughter is in fact good medicine (preventive or therapeutic) being associated with greater reduction in cortisol levels as compared with usual activities. These analyses demonstrated the potential therapeutic role of laughter-inducing interventions as a complementary strategy to improve everyone’s well-being and highlight the need for further research aiming to improve our collective sense of humor.

## Supporting information

S1 Checklist PRISMA 2020 checklist. (DOCX)

S1 Table Search strategies to identify interventional studies evaluating laughter interventions on cortisol levels. (DOCX)
