# Supplementary material for: Laughter as medicine: A systematic review and meta-analysis of interventional studies evaluating the impact of spontaneous laughter on cortisol levels
Source: PLoS One. 2023 May 23;18(5):e0286260. doi: 10.1371/journal.pone.0286260 (PMC10204943; doi:10.1371/journal.pone.0286260)
Supplement: S1 Table — (DOCX) [file pone.0286260.s002.docx]

| **Database/register** | **Controlled descriptors** | **Synonyms and/or keywords** | **Complete search strategy** |
| --- | --- | --- | --- |
| *MEDLINE/PubMed* | "Laughter"[Mesh] OR "Laughter therapy"[Mesh] | Humor therapy [Text Word] | ("Laughter"[Mesh] OR "Laughter therapy"[Mesh]) OR ("Laughter"[Text word] OR "Laughter therapy"[Text word]) OR (Humor therapy [Text Word]) |
| EMBASE | exp laughter therapy/ or laughter.mp. or exp laughter/ | Humor therapy.mp. | exp laughter therapy/ or laughter.mp. or exp laughter/ OR humor therapy.mp. |
| *PsycINFO* | Laughter | Humor | Laughter OR (humor) AND cortisol |
| Scopus | Laughter OR laughter therapy | Humor therapy | ( TITLE-ABS-KEY ( laughter )  OR  TITLE-ABS-KEY ( laughter  AND therapy ) OR    TITLE-ABS-KEY ( humor AND therapy)  AND  TITLE-ABS-KEY ( cortisol ) ) |
| Clinicaltrials.gov | Laughter | Humor | Laughter (or laughing) or humor in interventional studies |

**Online table 1.** Search strategies to identify interventional studies evaluating laughter interventions on

cortisol levels
